# Supplementary material for: Prediction of Primary Tumour and Axillary Lymph Node Response to Neoadjuvant Chemo(Targeted) Therapy with Dedicated Breast [18F]FDG PET/MRI in Breast Cancer
Source: Cancers (Basel). 2023 Jan 7;15(2):401. doi: 10.3390/cancers15020401 (PMC9857040; doi:10.3390/cancers15020401)
Supplement: Supplementary file 1 [file cancers-15-00401-s001.zip › cancers-2076823-supplementary.pdf]

cancers MDPI

Table S1. Administered NST regimens per breast cancer subtype with primary tumour and axillary pCR rates per regimen and subtype.

Subtype	Regimen	Primary tumour pCR		Axillary pCR	
**ER+/HER2-**	AC-docetaxel (*n=19*)	**2/19**	(11%)	5/13	(38%)
**ER+/HER2+**	AC-docetaxel and trastuzumab (*n=4*)	**3/4**	(75%)	0/0	(-)
	AC-docetaxel and trastuzumab/pertuzumab (*n=3*)	**3/3**	(100%)	3/3	(100%)
**ER-/HER2+**	AC-docetaxel and trastuzumab (*n=3*)	**2/3**	(67%)	1/1	(100%)
	AC-docetaxel and trastuzumab/pertuzumab (*n=3*)	**2/3**	(67%)	2/2	(100%)
**TNBC**	AC-paclitaxel (*n=10*)	4/10	(25%)	3/7	(42.9%)

Abbreviations: AC, doxorubicin and cyclophosphamide; ER, estrogen receptor; HER2, human epidermal growth factor receptor 2; pCR, pathologic complete response; TNBC, triple negative breast cancer.

Table S2. [18F]FDG PET/MRI protocol.

Sequence	Fat suppression	FOV (mm)	Voxel size (mm)	Slices	TR (ms)	TE (ms)	AT (s)	Turbo factor	Flip angle (degrees)
T2W	No	340	0.9 x 0.8 x 3.0	46	6410	83	328	11	80
DWI	Yes	320	1.7 x 1.7 x 4.0	24	-	-	-	-	-
T1W	Yes	340	0.9 x 0.9 x 1.2	128	4.77	1.78	541	-	10

Abbreviations: AT, acquisition time; DWI, diffusion weighted imaging; FOV, field of view; T1W, T1-weighted; T2W, T2-weighted; TE, echo time; TR, time repition.

The [18F]FDG PET/MR imaging protocol consisted of a two-dimensional T2-weighted (T2W) turbo spin-echo sequence without fat suppression, a diffusion weighted imaging (DWI) with b-values of 50, 400, 800, and 1000 s/mm^2 with fat suppression, a dynamic contrast enhanced (DCE)-T1-weighted (T1W) sequence with fat suppression and a fusion sequence of PET images with T1W and T2W images. For DWI, ADC parametric maps (in square millimetres per second) were created by using a linear least-squares fit of the log of the signal intensities at all b values (50, 400, 800, and 1000 s/mm2) and a classic monoexponential decay model. As a contrast agent for DCE-T1W, Gadobutrol (Gadovist ®, Bayer Health Care, Germany) was automatically injected through a catheter in the antecubital vein, with a dosage of 0.1 mmol/kg body weight, followed by a saline flush. Lastly, PET imaging consisted of an axial FOV of 258 mm. PET images were iteratively reconstructed by point spread function and automatic attenuation correction was applied using a 4-compartment (air, water, fat and lung adaptive) model attenuation map (Dixon-based μ-map). All PET images (single bed position) were acquired within 11 minutes of the initial activity measurement.

Table S3. Quantitative [18F]FDG PET/MR imaging variables in relation to pathological primary tumour response.

Variables	Primary tumour pCR		Primary tumour RD		P-value
Determined on primary tumour					
**LD (mm)**					
PETMRI-1	44.5	(13.0 to 68.0)	28.0	(9.0 to 72.0)	0.259
PETMRI-2	20.0	(0.0 to 46.0)	21.0	(6.0 to 65.0)	0.939
PETMRI-3	0.0	(0.0 to 37.0)	15.0	(0.0 to 38.0)	**0.018**
Δ2-1 (%)	-33.3	(-100.0 to -4.35)	-26.1	(-82.9 to 14.6)	0.077
Δ3-1 (%)	-100.0	(-100.0 to -19.57)	-40.9	(-100.0 to 0.0)	**0.012**
**SER**					
PETMRI-1	1.24	(1.14 to 1.47)	1.25	(0.75 to 1.54)	0.484
PETMRI-2	0.89	(0.43 to 1.44)	1.07	(0.39 to 1.80)	0.128
PETMRI-3	0.61	(0.28 to 1.03)	0.71	(0.45 to 1.30)	0.061
Δ2-1 (%)	-30.1	(-68.0 to 8.0)	-13.0	(-69.5 to 46.5)	**0.044**
Δ3-1 (%)	-54.3	(-75.4 to -14.5)	-38.4	(-65.3 to 0.6)	**0.013**
**Mean ADC**					
PETMRI-1	953	(788 to 1369)	913	(643 to 1288)	0.338
PETMRI-2	1536	(745 to 1882)	1226	(914 to 1998)	0.063
PETMRI-3	1362	(979 to 2058)	1461	(835 to 2200)	0.688
Δ2-1 (%)	46.4	(-15.3 to 119.0)	41.9	(-7.8 to 79.2)	0.678
Δ3-1 (%)	51.2	(-6.6 to 151.9)	47.5	(-8.3 to 117.7)	0.760
**Min ADC**					
PETMRI-1	643	(410 to 811)	583	(288 to 951)	0.074
PETMRI-2	789	(108 to 1355)	785	(226 to 1184)	0.902
PETMRI-3	762	(198 to 1673)	952	(250 to 1518)	0.386
Δ2-1 (%)	25.2	(-84.3 to 182.9)	39.4	(-59.8 to 161.3)	0.451
Δ3-1 (%)	31.7	(-71.2 to 237.3)	51.6	(-17.2 to 241.9)	0.297
**SUV_{max}**					
PETMRI-1	8.8	(1.0 to 21.9)	4.8	(1.1 to 16.0)	0.312
PETMRI-2	1.7	(0.4 to 3.1)	2.0	(0.8 to 8.1)	0.196
PETMRI-3	1.2	(0.3 to 1.9)	1.3	(0.4 to 7.0)	0.232
Δ2-1 (%)	-82.6	(-94.1 to -9.2)	-40.7	(-87.7 to 8.9)	**0.017**
Δ3-1 (%)	-84.1	(-96.3 to -28.1)	-69.2	(-91.8 to -29.2)	0.098
**SUV_{peak}**					
PETMRI-1	6.4	(0.6 to 17.3)	3.6	(0.8 to 11.7)	0.393
PETMRI-2	1.3	(0.3 to 1.9)	1.7	(0.6 to 6.3)	0.186
PETMRI-3	1.0	(0.3 to 1.6)	1.1	(0.3 to 4.2)	0.263
Δ2-1 (%)	-78.7	(-90.9 to 2.4)	-39.7	(-89.7 to 12.2)	**0.026**
Δ3-1 (%)	-81.5	(-96.3 to -18.7)	-67.0	(-89.9 to -28.4)	0.187
**$SUV_{30\%}$**					
PETMRI-1	4.4	(0.5 to 11.3)	2.3	(0.6 to 8.6)	0.282
PETMRI-2	1.0	(0.2 to 1.6)	1.1	(0.4 to 4.3)	0.389
PETMRI-3	0.8	(0.2 to 1.0)	0.8	(0.2 to 3.1)	0.214
Δ2-1 (%)	-77.9	(-93.5 to 6.9)	-38.9	(-89.4 to 16.2)	**0.022**
Δ3-1 (%)	-81.3	(-97.1 to -17.6)	-61.2	(-89.4 to -25.7)	0.198
**$MTV_{30\%}$**					
PETMRI-1	8.4	(2.1 to 30.3)	6.8	(1.8 to 54.1)	0.816
PETMRI-2	9.4	(0.9 to 23.0)	5.3	(1.8 to 36.4)	0.926
PETMRI-3	4.6	(0.3 to 16.7)	4.8	(0.3 to 22.6)	0.759
Δ2-1 (%)	37.3	(-90.7 to 123.5)	-20.4	(-53.8 to 224.9)	0.633
Δ3-1 (%)	-31.8	(-96.6 to 271.8)	-27.5	(-92.6 to 313.6)	0.581

Table S3. *Continued.*

**$TLG_{30\%}$**					
PETMRI-1	26.7	(1.3 to 240.8)	16.2	(3.1 to 109.3)	0.223
PETMRI-2	8.0	(0.6 to 22.6)	7.2	(1.0 to 53.6)	0.451
PETMRI-3	2.8	(0.1 to 17.1)	5.1	(0.2 to 27.4)	0.257
Δ2-1 (%)	-62.5	(-99.4 to 93.1)	-44.7	(-94.4 to 92.8)	0.077
Δ3-1 (%)	-88.1	(-99.8 to 25.6)	-73.1	(-98.2 to 19.7)	0.125
**$SUV_{40\%}$**					
PETMRI-1	5.2	(0.6 to 13.1)	2.6	(0.6 to 10.0)	0.265
PETMRI-2	1.1	(0.2 to 1.8)	1.2	(0.4 to 4.9)	0.268
PETMRI-3	0.8	(0.3 to 1.1)	0.9	(0.2 to 3.9)	0.187
Δ2-1 (%)	-80.1	(-94.1 to -1.8)	-39.5	(-89.7 to 12.4)	**0.019**
Δ3-1 (%)	-83.4	(-96.9 to -19.8)	-64.1	(-90.9 to -27.4)	0.158
**$MTV_{40\%}$**					
PETMRI-1	5.9	(1.4 to 21.9)	4.4	(1.1 to 38.2)	0.796
PETMRI-2	7.4	(0.9 to 15.1)	4.1	(1.1 to 32.2)	0.678
PETMRI-3	4.2	(0.3 to 13.0)	3.8	(0.3 to 20.4)	0.724
Δ2-1 (%)	64.3	(-89.4 to 199.6)	-11.7	(-54.4 to 322.4)	0.633
Δ3-1 (%)	-25.9	(-95.7 to 193.6)	-32.3	(-89.9 to 469.1)	0.646
**$TLG_{40\%}$**					
PETMRI-1	21.7	(0.9 to 197.9)	11.2	(2.6 to 85.6)	0.244
PETMRI-2	6.7	(0.5 to 18.4)	5.5	(0.8 to 49.8)	0.612
PETMRI-3	2.4	(0.1 to 14.7)	4.5	(0.2 to 19.8)	0.283
Δ2-1 (%)	-60.6	(-99.3 to 143.6)	-36.3	(-94.6 to 101.3)	0.128
Δ3-1 (%)	-87.4	(-99.7 to 0.9)	-74.5	(-97.8 to 19.7)	0.104
**$SUV_{50\%}$**					
PETMRI-1	5.9	(0.7 to 14.9)	3.0	(0.7 to 11.6)	0.277
PETMRI-2	1.1	(0.2 to 2.0)	1.3	(0.5 to 5.5)	0.213
PETMRI-3	0.9	(0.3 to 1.2)	0.9	(0.2 to 4.7)	0.263
Δ2-1 (%)	-81.9	(-94.3 to -4.3)	-40.4	(-89.3 to 11.9)	**0.019**
Δ3-1 (%)	-84.3	(-96.6 to -19.8)	-67.6	(-92.0 to -27.1)	0.111
**$MTV_{50\%}$**					
PETMRI-1	3.6	(0.9 to 16.4)	2.9	(0.6 to 19.8)	0.660
PETMRI-2	5.6	(0.7 to 11.5)	3.1	(0.6 to 23.6)	0.406
PETMRI-3	2.9	(0.3 to 9.8)	2.9	(0.3 to 17.8)	0.794
Δ2-1 (%)	63.5	(-87.7 to 305.3)	14.7	(-65.5 to 319.7)	0.489
Δ3-1 (%)	-30.4	(-95.3 to 245.2)	-11.8	(-85.6 629.7)	0.425
**$TLG_{50\%}$**					
PETMRI-1	15.9	(0.6 to 161.5)	7.5	(2.2 to 69.1)	0.265
PETMRI-2	5.5	(0.5 to 16.9)	4.1	(0.5 to 39.9)	0.914
PETMRI-3	2.0	(0.1 to 12.0)	3.7	(0.2 to 16.1)	0.312
Δ2-1 (%)	-50.6	(-99.3 to 215.0)	-30.7	(-95.8 to 104.8)	0.113
Δ3-1 (%)	-85.5	(-99.7 to 49.7)	-69.1	(-97.1 to 20.0)	0.111

Quantitative imaging variables are compared between primary tumour pCR and RD by means of Mann-Whitney U test. Abbreviations: ADC, apparent diffusion coefficient; LD, longest diameter; MTV, metabolic tumour volume; pCR, pathologic complete response; RD, residual disease; SER, signal enhancement ratio; SUV, standardized uptake value; TLG, total lesion glycolysis.

Table S4. Quantitative [18F]FDG PET/MR imaging variables determined on the primary tumour as well as on the most FDG-avid axillary lymph node in relation to pathological axillary response.

Variables	Axillary pCR		Axillary RD		P-value
Determined on primary tumour					
**LD**					
PETMRI-1 (mm)	37.5	(13.0 to 68.0)	33.0	(15.0 to 70.0)	0.589
PETMRI-2 (mm)	24.0	(0.0 to 65.0)	17.0	(6.0 to 47.0)	0.895
PETMRI-3 (mm)	0.0	(0.0 to 38.0)	15.0	(0.0 to 30.0)	**0.047**
Δ2-1 (%)	-33.7	(-100.0 to -4.4)	-34.4	(-82.9 to 14.6)	0.391
Δ3-1 (%)	-100.0	(-100.0 to -28.3)	-53.8	(-100.0 to 0.0)	**0.026**
Determined on most FDG-avid axillary lymph node					
**SUV_{max}**					
PETMRI-1	4.7	(0.6 to 21.7)	2.6	(1.3 to 15.9)	0.837
PETMRI-2	0.5	(0.4 to 2.7)	0.9	(0.6 to 5.5)	**0.030**
PETMRI-3	0.5	(0.3 to 1.1)	0.6	(0.2 to 1.3)	0.654
Δ2-1 (%)	-88.0	(-96.1 to -37.3)	-59.8	(-93.6 to -7.3)	**0.010**
Δ3-1 (%)	-89.7	(-97.5 to 14.7)	-72.7	(-95.4 to -53.0)	0.233
**SUV_{peak}**					
PETMRI-1	2.5	(0.5 to 16.6)	1.9	(1.0 to 13.4)	0.959
PETMRI-2	0.5	(0.4 to 1.7)	0.8	(0.6 to 4.2)	**0.010**
PETMRI-3	0.5	(0.2 to 1.1)	0.6	(0.2 to 1.2)	0.575
Δ2-1 (%)	-84.7	(-95.9 to -28.0)	-56.9	(-93.4 to -8.4)	**0.030**
Δ3-1 (%)	-84.9	(-96.8 to 54.4)	-68.3	(-94.5 to -40.4)	0.412
Determined on most FDG-avid part primary tumour and most FDG-avid axillary lymph node					
**NT-ratio**					
PETMRI-1	0.9	(0.1 to 5.8)	1.0	(0.4 to 3.5)	0.527
PETMRI-2	0.4	(0.2 to 1.5)	0.6	(0.2 to 3.8)	**0.041**
PETMRI-3	0.4	(0.2 to 2.0)	0.6	(0.2 to 0.7)	0.941
Δ2-1 (%)	-59.7	(-92.6 to 42.3)	-35.7	(-66.0 to 78.1)	**0.018**
Δ3-1 (%)	-61.6	(-80.4 to 152.9)	-35.2	(-78.5 to 64.9)	0.823

Quantitative imaging variables are compared between primary tumour pCR and RD by means of Mann-Whitney U test. Abbreviations: LD, longest diameter; NT-ratio, nodal-to-tumour ratio; pCR, pathologic complete response; RD, residual disease; SUV, standardized uptake value.
